# SIV Vpx Is Essential for Macrophage Infection but Not for Development of AIDS

**DOI:** 10.1371/journal.pone.0084463

**Published:** 2014-01-21

**Authors:** Susan V. Westmoreland, A. Peter Converse, Kasia Hrecka, Mollie Hurley, Heather Knight, Michael Piatak, Jeffrey Lifson, Keith G. Mansfield, Jacek Skowronski, Ronald C. Desrosiers

**Affiliations:** 1 Harvard Medical School, New England Primate Research Center, Division of Comparative Pathology, Southborough, Massachusetts, United States of America; 2 AIDS and Cancer Virus Program NCI-Frederick, SAIC-Frederick, Frederick, Maryland, United States of America; 3 Case Western Reserve University School of Medicine, Department of Molecular Biology and Microbiology, Cleveland, Ohio, United States of America; 4 Harvard Medical School, New England Primate Research Center, Division of Microbiology, Southborough, Massachusetts, United States of America; University of Pittsburgh Center for Vaccine Research, United States of America

## Abstract

Analysis of rhesus macaques infected with a *vpx* deletion mutant virus of simian immunodeficiency virus mac239 (SIVΔ*vpx*) demonstrates that Vpx is essential for efficient monocyte/macrophage infection *in vivo* but is not necessary for development of AIDS. To compare myeloid-lineage cell infection in monkeys infected with SIVΔ*vpx* compared to SIVmac239, we analyzed lymphoid and gastrointestinal tissues from SIVΔ*vpx*-infected rhesus (n = 5), SIVmac239-infected rhesus with SIV encephalitis (7 SIV239E), those without encephalitis (4 SIV239noE), and other SIV mutant viruses with low viral loads (4 SIVΔ*nef*, 2 SIVΔ3). SIV+ macrophages and the percentage of total SIV+ cells that were macrophages in spleen and lymph nodes were significantly lower in rhesus infected with SIVΔ*vpx* (2.2%) compared to those infected with SIV239E (22.7%), SIV239noE (8.2%), and SIV mutant viruses (10.1%). In colon, SIVΔ*vpx* monkeys had fewer SIV+ cells, no SIV+ macrophages, and lower percentage of SIV+ cells that were macrophages than the other 3 groups. Only 2 SIVΔ*vpx* monkeys exhibited detectable virus in the colon. We demonstrate that Vpx is essential for efficient macrophage infection *in vivo* and that simian AIDS and death can occur in the absence of detectable macrophage infection.

## Introduction

While all lineages of HIV and SIV encode an accessory protein termed viral protein R (Vpr), only some, particularly the HIV-2, SIV_SM_, and SIV_MAC_ lineages, encode viral protein X (Vpx) [1]–[3]. Based on sequence similarity to the *vpr* gene, it has been theorized that *vpx* arose as a duplication of an ancestral *vpr* [3] or as a result of a recombination event [3], [4]. Despite the recognition that Vpx and Vpr have distinct functions [5], they both have been reported to play a role in viral replication in macrophages and dendritic cells [2], [6]–[11]. While Vpr may enhance viral replication in myeloid-lineage cells in vitro [6], the effect of Vpx on replication in myeloid cells is even more dramatic [6], [7]. Recently it has been shown that Vpx targets for ubiquitin-mediated proteasomal degradation a cellular restriction factor, SAM domain HD domain-containing protein 1 (SAMHD1) [8], [9], [12]–[14]. SAMHD1 would otherwise inhibit viral infection of myeloid and dendritic cells by depleting deoxynucleotide triphosphates (dNTPs) needed by the lentivirus to complete reverse transcription [9]. SAMHD1 also restricts HIV-1 infection of resting CD4 cells [15], [16]. Through mechanisms still under examination, SAMHD1 is also able to restrict replication of virus transmitted by cell-to-cell contact to monocyte-derived dendritic cells (MDDCs) [17]. Despite the relatively low infection rate of myeloid and dendritic cells by HIV-1, examining the role of Vpx in these cells it is important to understand their role of HIV infection [18].

Although recent studies have elucidated Vpx function *in vitro*, there is limited information defining the role and impact of Vpx *in vivo*. Two early studies were conducted with Vpx mutant viruses, one study with mucosal inoculation of pigtail macaques (*Macaca nemestrina*) using a SIV_SM_PBj6.6 *vpx*-I activated mutant virus [2] and one in rhesus macaques (*Macaca mulatta*) infected intravenously with a *vpx* deletion mutant of SIVmac239 (SIVΔ*vpx*) [19]–[21]. Viral replication and dissemination of the *vpx*-mutant SIV_SM_ during acute infection were significantly hindered [2]. Likewise, the extent of SIVΔ*vpx* replication was diminished relative to the parental wild type virus and chronic infection and survival was prolonged [19]–[21].

In this study, we analyzed the cellular and tissue targets of viral replication in adult rhesus monkeys infected with SIVΔ*vpx* after chronic infection at terminal AIDS. The SIVΔ*vpx* mutant virus is identical to SIVmac239 wild-type cloned virus except for the 101 base deletion of the *vpx* gene from the virus. In previous studies, we demonstrated that SIVΔ*vpx* replicates in rhesus PBMC with only slightly reduced kinetics, but its replicates in rhesus macrophages was markedly impaired with no significant replication noted over 30 days [19].

Cellular and tissue tropism of SIVΔ*vpx*-infected macaques were compared to those of rhesus infected with SIVmac239 with (SIV239E) or without encephalitis (SIV239noE), SIVΔ*nef*, or SIVΔ*3*. SIVΔ*3* is missing *nef*, *vpr*, and upstream sequences in *U3* [22]. We demonstrate that lack of *vpx* function results in the near complete absence of infection in myeloid-lineage cells *in vivo* even after several years. Additionally, the colon is particularly devoid of virus despite the presence of remaining lymphoid cells. Nevertheless, all 5 SIVΔ*vpx*-inoculated rhesus developed AIDS with opportunistic infections and AIDS-defining lesions [20]. Taken together, these findings indicate that Vpx is required for efficient infection of myeloid-lineage cells *in vivo* and that the development of AIDS can occur in the absence of detectable virus replication in myeloid-lineage cells, including macrophages and dendritic cells.

## Results

### SIVΔ*vpx*-infected rhesus monkeys develop AIDS with prolonged survival and no encephalitis

**Figure 1:**
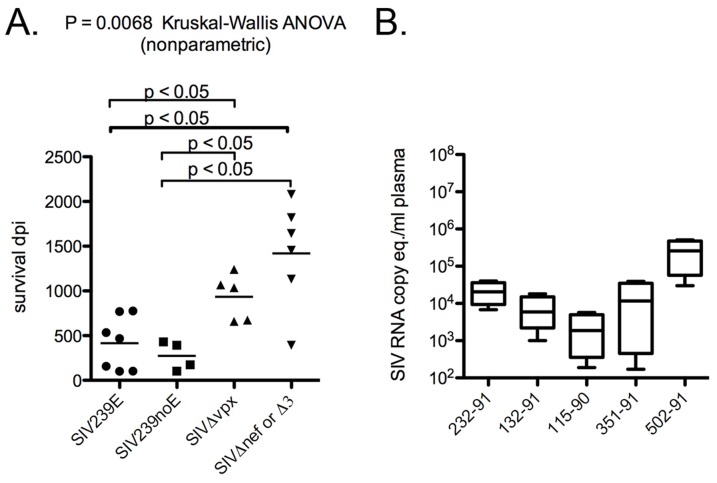
Survival and viral load data. (**A)** Survival days post-inoculation (dpi) for rhesus macaques inoculated with SIVΔ*vpx* compared to animals inoculated with SIVmac239 (SIV239E with encephalitis or SIV239noE without encephalitis) or other mutant viruses (SIVΔ*nef* or SIVΔ3. SIVΔ*vpx*-infected macaques survived significantly longer with a slower disease progression compared to SIV239E or SIV239noE animals, but did not differ in survival length compared to SIVΔ*nef* or SIVΔ3. **(B)** Plasma viral RNA from SIVΔ*vpx*-infected rhesus macaques expressed as RNA copy equivalents per ml plasma from chronic disease or near-terminal collections (range 329–1140 dpi, median 730 dpi).

The initial infection of five rhesus macaques with SIVΔ*vpx* was described previously by Gibbs et al. [19], [20]. Survival of these SIVΔ*vpx*-infected rhesus ranged from 659 to 1241 days post-inoculation (dpi) with median survival of 1036 dpi (**Table 1**). SIVΔ*vpx*-infected monkeys had significantly longer survival (Kruskal-Wallis ANOVA, p = 0.0068) compared to both SIV239E and SIV239noE monkeys (median survival 438 dpi) (**Figure 1a**). All 5 SIVΔ*vpx*-infected rhesus developed AIDS with AIDS-defining lesions including opportunistic infections (**Table 2**), while none developed multinucleated giant cell disease (GCD) or SIV giant cell encephalitis (SIVE), both disease manifestations of lentiviral infection characterized by histologically distinctive infected macrophages [23]–[25].

**Table 1:** Study cohort of SIVΔ*vpx* (n = 5) and SIVmac239 (n = 11) with and without encephalitis.

Inoculum	Case #	Survival (days)	AIDS	SIVE
SIVΔ*vpx*	**1**	659	Y	N
SIVΔ*vpx*	**2**	673	Y	N
SIVΔ*vpx*	**3**	1036	Y	N
SIVΔ*vpx*	**4**	1068	Y	N
SIVΔ*vpx*	**5**	1241	Y	N
		mean 935.4/median 1036		
SIV239E	**6**	100	Y	Y
SIVmac239	**7**	103	Y	Y
SIVmac239	**8**	157	Y	Y
SIVmac239	**9**	467	Y	Y
SIVmac239	**10**	535	Y	Y
SIVmac239	**11**	769	Y	Y
SIVmac239	**12**	777	Y	Y
SIVmac239	**13**	103	Y	N
SIVmac239	**14**	173	Y	N
SIVmac239	**15**	392	Y	N
SIVmac239	**16**	431	Y	N
		mean 364.3/median 438.5		
SIVΔ*nef*	**17**	392	Y	N
SIVΔ*nef*	**18**	1456	Y	N
SIVΔ*nef*	**19**	1642	Y	N
SIVΔ*nef*	**20**	2080	Y	N
SIVΔ3	**21**	1132	Y	N
SIVΔ3	**22**	1820	Y	N
		Mean 1420/median 1549		

**Table 2:** SIVΔ*vpx* systemic and neuropathologic findings associated with terminal AIDS.

Case #	Path #	Morphologic Diagnosis
**1**	A93-673	Wasting with emaciation; bacterial enterocolitis; invasive trichomoniasis
**2**	A93-695	Adenoviral colitis; SIV arteriopathy; lymphoplasmacytic choroid plexitis
**3**	A94-594	Adenoviral gastritis; Pneumocystis pneumonia; intestinal trichomoniasis
**4**	A96-85	Cytomegalovirus, disseminated; Mycobacteriosis, disseminated; Pneumocystis pneumonia; glomerulonephritis; granulomatous hepatitis
**5**	A96-368	Pneumocystis pneumonia; intestinal Mycobacteriosis; Cytomegalovirus myelitis, meningitis; severe gastritis

### SIVΔ*vpx* plasma viral loads

The original analysis of these SIVΔ*vpx*-infected monkeys revealed lower virus burdens with more PBMCs required to recover virus in CEMx174 co-cultures compared to SIVmac239 wild-type infected rhesus [20]. To compare plasma viral RNA levels using current techniques, we used quantitative RT-PCR to analyze frozen plasma samples from monkeys chronically infected by SIVΔ*vpx* (**Figure 1b** **)** and compared the levels to the well-documented levels in SIVmac239-infected rhesus. Viral RNA levels in SIVΔ*vpx*-infected rhesus from the chronic phase of infection ranged from 2.9×10^3^ to 5.1×10^5^ (mean 1.1×10^5^, median 1.7×10^4^ RNA copy eq/ml plasma), which are 0.5-2.5 logs lower than usually observed in SIVmac239-infected rhesus [20], [26]–[28]. SIVΔ*vpx* case 4 had the highest near-terminal plasma viral load (5.1×10^5^), but still survived 1068 dpi (almost 3 years) before succumbing to AIDS. SIVΔ*nef* and SIVΔ3 infected rhesus had chronic and near-terminal plasma viral loads comparable to SIVΔ*vp*x cases, as previously reported [29].

### Viral sequences in SIVΔ*vpx*-infected monkeys

**Figure 2:**
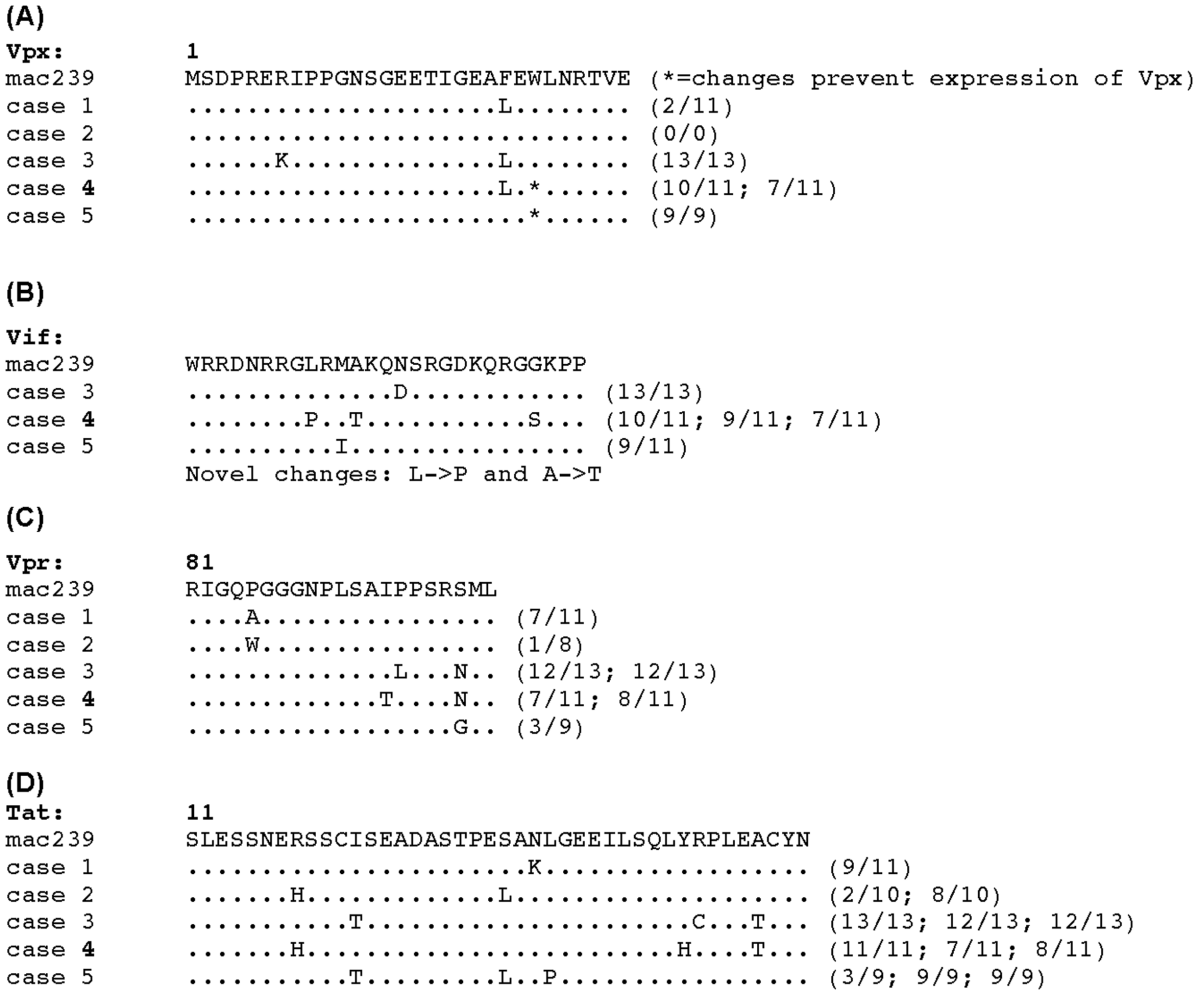
Amino acid sequences from chronic infection plasma viral RNA from SIVΔ*vpx*-infected monkeys. **(A).** Vpx N-terminal sequences accumulated debilitating mutations in two animals (cases 4 and 5) (*). **(B).** Novel amino acid sequence changes of Vif were present in case 4 (with highest viral loads) with leucine to proline (L->P) and alanine to threonine (A->T). **(C).** Analysis of Vpr revealed changes in amino acid sequences detected at the C-terminal region in cases 3 and 4 (I94T, P95L, S99N or G). **(D).** Alignments of Tat demonstrated multiple non-conservative changes, such as I22T, S32L, L35P.

The *vpx-vpr* region was amplified from frozen plasma samples available from the SIVΔ*vpx*-infected rhesus macaques. Alignments of the predicted amino acid sequences for Vpx, Vpr, and overlapping Vif and Tat segments were constructed. The original 101 base pair deletion in *vpx* eliminated Vpx function measured in vitro and was carefully constructed so as not to affect the overlapping Vif sequences at its amino terminus or the splice acceptor for Vpr near the C terminus [19], [20]. The deletion was intentionally made out-of-frame such that *vpx* sequences downstream of the deletion would be out-of-frame with stop codons immediately flanking the deletion [19], [20]. Alignment of sequences obtained from the plasma samples taken near the time of death revealed, as expected, consistent preservation of the original 101 bp deletion, which spans the Cullin 4 E3 binding region required for counteraction of SAMHD1 and efficient myeloid cell infection *in vitro* (**Figure S1 a, b**). Sequences from 2 of the five animals (cases 4 and 5) revealed additional stop codons in the remaining *vpx* sequences (**Figure 2**), consistent with virus no longer needing to retain the coding capacity for the *vpx* sequences that remained [30]. No consistent patterns of sequence changes were observed in the portions examined of Vpx, Vpr, Vif or Tat from the five monkeys (**Figure 2**). However, Vif sequences in the monkey with the highest viral loads (#4) did exhibit two novel (not seen in the Los Alamos Compendium of sequences) amino acid changes (L->P and A->T).

### Tissue virus burden and cellular targets

**Figure 3:**
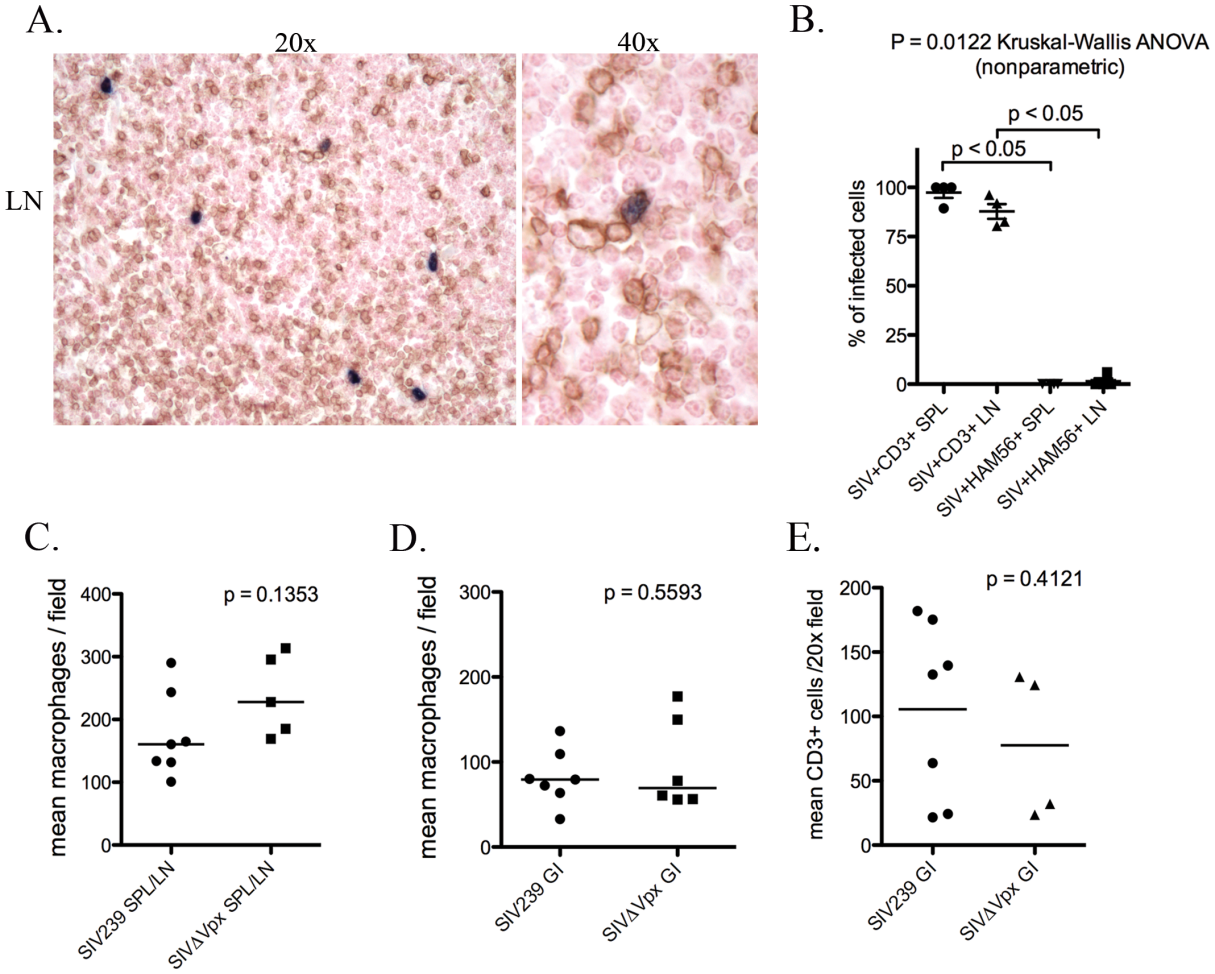
Quantification of tissue macrophages and lymphocytes in SIVΔ*vpx* compared to SIVmac239-infected rhesus. **(A).** Rhesus, lymph node: representative image of SIV *in situ* hybridization (ISH, blue) with double-label immunohistochemistry for CD3+ T lymphocytes (DAB, brown); **(B).** Quantification of immunophenotyped SIVΔ*vpx*-infected cells demonstrates almost exclusive infection of CD3+ T lymphocytes and not Ham56+ macrophages in spleen and lymph nodes (p<0.05); **(C).** Numbers of Ham56+ tissue macrophages in the spleen or lymph node and colon were not significantly different in SIVΔ*vpx*-infected rhesus macaques compared to SIVmac239-infected rhesus; **(D).** Numbers of CD3+ lymphocytes were not significantly different in the colon in SIVΔ*vpx*-infected rhesus macaques compared to SIVmac239-infected rhesus.

To assess the presence of cellular targets for viral infection in spleen, lymph node and colon, immunohistochemistry was used to identify Ham56+ myeloid cells and CD3+ T lymphocytes. The overall number of Ham56+ myeloid cells (including macrophages and dendritic cells) did not differ between SIVΔ*vpx*-infected monkeys compared to rhesus infected with SIVmac239 in the spleen, lymph node, or colon (**Figure 3A, B**). In addition, there was no significant difference in the presence of CD3+ T lymphocytes in the lamina propria of the colon between SIVmac239 versus SIVΔ*vpx*-infected monkeys (**Figure 3C**).

**Figure 4:**
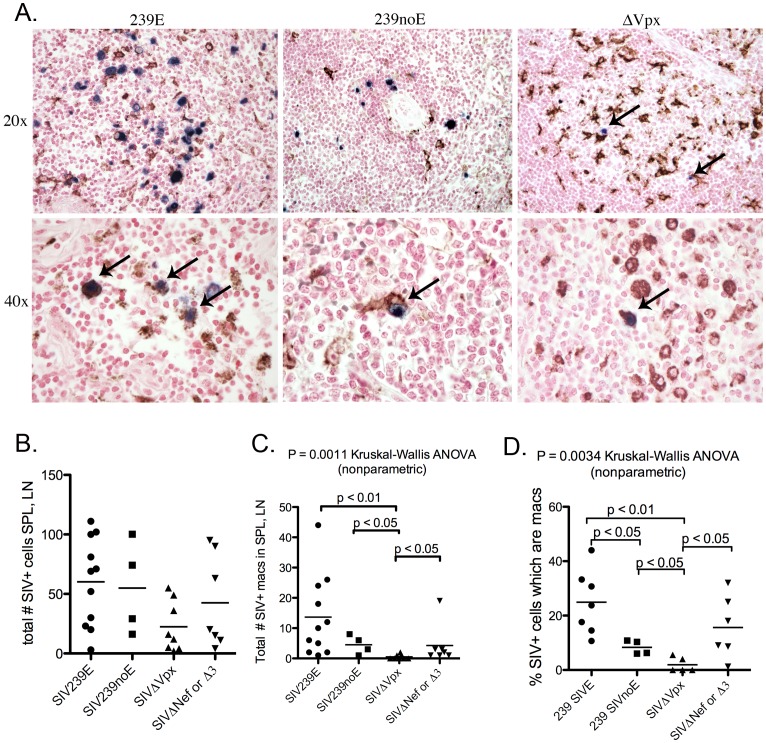
Double-label immunohistochemistry and *in situ* hybridization of lymphoid tissues. **(A)** Representative images of SIV *in situ* hybridization (ISH, blue) with double-label immunohistochemistry for monocyte/macrophage lineage cell marker Ham56 (DAB, brown) in rhesus macaques in groups SIV239E (left), SIV239noE (middle), and SIVΔ*vpx* (right). The top images of lymphoid follicles depict Ham-56+ cells morphologically consistent with follicular DCs. Images in the second row depict HAM56+ cells in the red pulp of the spleen (left and center) or paracortex in lymph node (right). Frequent SIV+ HAM56+ macrophages/DCs (Mφ) were observed in tissues from the SIV239E and SIV239noE groups, while only rare SIV+ Ham56+ macrophages/DCs were observed in lymph node from a SIVΔ*vpx*-infected rhesus (arrow). **(B).** The overall numbers of infected cells in the spleen and lymph node from the 4 groups of animals were not significantly different. **(C)** However, there were significantly fewer SIV+ Mφ in SIVΔ*vpx* monkeys (mean 0.5 SIV+ Mφ) compared to the SIV239E (mean 13.64 SIV+ Mφ), SIV239noE (mean 4.5 SIV+ Mφ) and SIVΔ*nef*/SIVΔ3 monkeys (mean 6.25 SIV+ Mφ). **(D)** SIV infected macrophages made up a much lower percentage of all SIV+ cells in SPL and LN of SIVΔ*vpx*-infected rhesus (mean 2.2%) compared to SIV239E (mean 22.7%), SIV239noE (mean 8.3%), and SIVΔ*nef*/SIVΔ3 monkeys (10.1%) (p<0.05).

To analyze the impact of deletion of *vpx* on tissue virus burden and tissue tropism, we compared the numbers of SIV+ cells in available spleen (SPL), peripheral lymph node (LN), and colon tissues (all when available) among the 4 groups of monkeys. Photomicrographs of representative sections of *in situ* labeled SIV+ cells in spleen and lymph node depict high levels of infection in the SIV239E and SIV239noE monkeys compared to the SIVΔ*vpx* cases (**Figure 4A**). Total number of infected cells per sections of SPL and LN showed that there was no significant difference between the groups (ANOVA, p = 0.0706), but there was a trend for the highest amount of virus in the SIV239E group (mean 60.15 SIV+ cells), followed by the SIV239noE group (mean 54.75 SIV+ cells), and the SIVΔ*nef*/SIVΔ3 group (mean 42.5 SIV+ cells), with the least in the SIVΔ*vpx* group (mean 23.5 SIV+ cells) (**Figure 4B**). Consistent with the plasma viral load data, the combined number of infected cells in lymphoid tissues (spleen and lymph node) among the *SIV*Δ*vpx* cases was highest in case #4 (**Figure 1b**).

**Figure 5:**
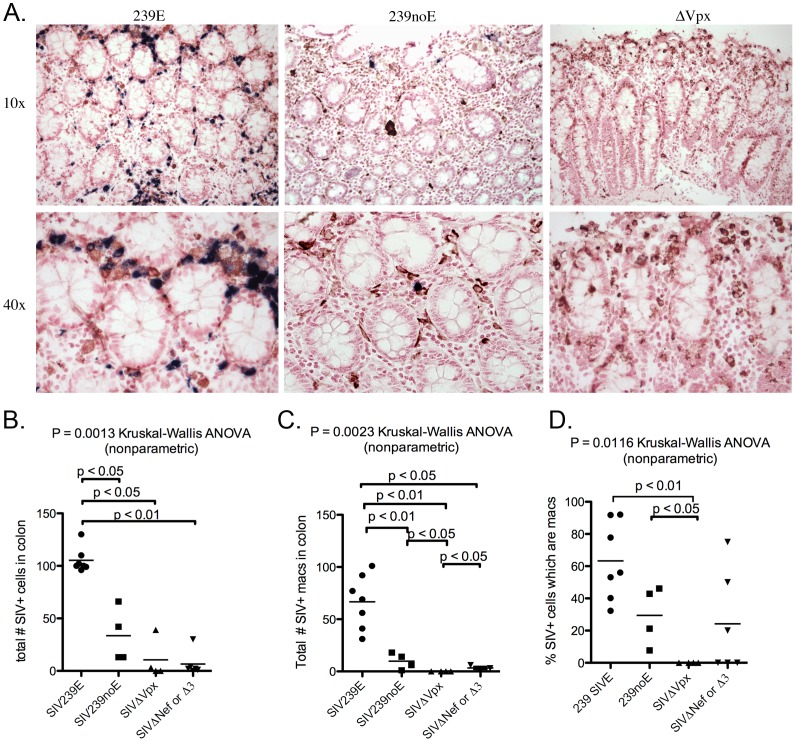
Double-label immunohistochemistry and *in situ* hybridization of colon. **(A).** Representative images of SIV ISH (blue) with double-label immunohistochemistry for macrophage marker Ham56 (DAB, brown) in rhesus macaques in groups SIV239E (left), SIV239noE (middle), and SIVΔ*vpx* (right). SIV+ Ham56+ macrophages were frequent in SIV239E monkeys (left), but absent in the SIVΔ*vpx* group (right). **(B).** SIVΔ*vpx*-infected monkeys had significantly less virus in the colon (mean 10.5 SIV+ cells) compared to monkeys infected with SIV239 (SIV239E mean 105.3 SIV+ cells, SIV239noE mean 33.5 SIV+ cells), but no difference with animals in the SIVΔ*nef*/SIVΔ3 group. **(C).** SIVΔ*vpx* monkeys (mean 0 cells) had significantly fewer SIV+ Ham56+ macrophages compared to SIV239E (mean 66.7 cells), SIV239noE (mean 9.8 cells), and SIVΔ*nef*/SIVΔ3 groups (mean 3.3 cells) groups. **(D).** There was a significantly lower percentage of SIV+ cells that were macrophages in SIVΔ*vpx*-infected rhesus (mean 0%) compared to both the SIV239E (mean 63.3%) and SIV239noE (mean 22.4%) groups and a trend in the SIVΔ*nef*/SIVΔ3 group (mean 25.6%).

The number of SIV infected cells in the colon differed significantly among the 4 groups (ANOVA, p = 0.0013) (**Figure 5A, B**). The mean of SIV+ cells in the colon was significantly lower in the SIVΔ*vpx*-infected monkeys (mean 10.5) and the SIVΔ*nef*/SIVΔ3 group (mean 6.5) compared to both the SIV239E (mean 105.3) and the SIV239noE monkeys (mean 33.5) (p<0.05) (**Figure 5B**), but did not differ significantly between the SIVΔ*vpx* and SIVΔ*nef*/SIVΔ3 animals. Only 2 of the 5 SIVΔ*vpx* cases (cases 1 and 4) had detectable SIV+ cells in the colon, whereas 4/5 SIVΔ*nef*/SIVΔ3 infected rhesus had detectable virus in the colon. In addition, virus was abundant in SIV239E cases and frequent in SIV239noE cases (**Figure 5B**). The dearth of SIV in the colon of SIVΔ*vpx*-infected monkeys was not due to a lack of target lymphocytes and macrophages as both cell types were abundant at levels not significantly different from SIVmac239-infected monkeys (**Figure 3B, C**).

### Quantification of SIV infected cell types *in vivo*

**Figure 6:**
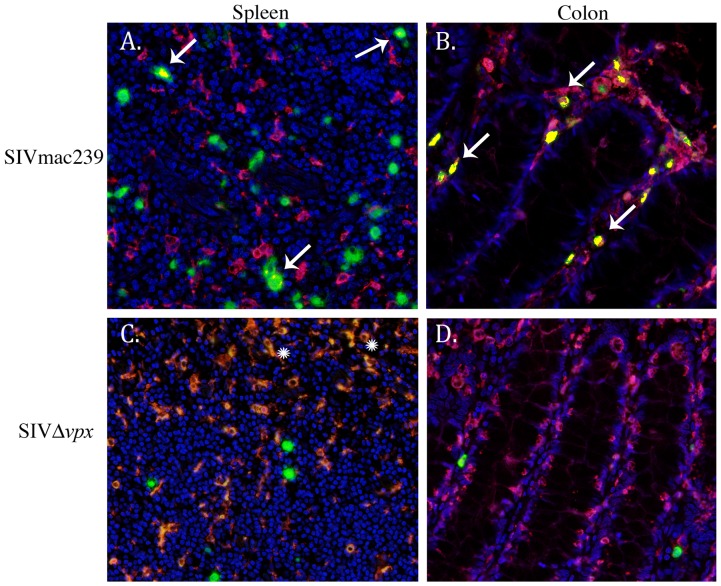
Spectral imaging and colocalization of double-label immunohistochemistry slides of spleen and colon. Immunofluorescence with SIV nucleic acid (green), Ham56+ macrophages (red), and coexpression (yellow, arrows) demonstrate that infected macrophages are readily evident in SIVmac239-infected monkeys (**A, B, arrows**), particularly within the colon (**B**), but are absent in spleen and colon from SIVΔ*vpx*-infected monkeys (**C, D**). Snowflake symbols in **C** denote autofluorescence in red pulp macrophages. Original magnification 40× (**A**–**D**).

We analyzed the available SPL, LN, and colon tissues from monkeys in all groups using double-label SIV *in situ* hybridization and Ham-56 immunohistochemistry. Images of lymphoid follicles (top row) depict Ham-56+ cells morphologically consistent with follicular DCs, while images in the second row depict HAM56+ macrophage/dendritic cells in the red pulp of the spleen (left and center) or paracortex in lymph node (right) (**Figure 4** **A**). Although the total number of SIV+ cells in SPL and LN did not differ significantly among the 4 groups, the total number of infected myeloid-lineage cells, including macrophages and dendritic cells, did differ among the groups (ANOVA p = 0.0011) with significantly higher SIV+ macrophage mean in the SIV239E group (mean 13.6 cells) and SIV239noE (mean 4.5 cells) compared to SIVΔ*vpx* monkeys (mean 0.5 cells) (P<0.05 for both) (**Figure 4C**). Of importance is the observation that the number of SIV+ myeloid cells in the SPL/LN tissues from rhesus in the SIVΔ*nef*/SIVΔ3 group was significantly higher (p<0.05) than in the SIVΔ*vpx* monkeys while there was no difference in the overall number of infected cells in these tissues (**Figure 4** **B, C**). SIV infected macrophages made up a much higher percentage of all SIV+ cells in SPL and LN of SIV239E (22.7%), SIV239noE (8.3%), and SIVΔ*nef*/SIVΔ3 monkeys (10.1%) compared to SIVΔ*vpx* (2.2%) (p<0.05) (**Figure 4D**). This is supported by additional assays demonstrating that 80.4–100% of SIV+ cells in the SPL and LN from SIVΔ*vpx* monkeys were definitively identified as CD3+ T cells (**Figure 3D**). Overall, only 4 infected macrophages were detected out of 179 SIV+ cells (2.2%) counted in all SPL and LN sections from the 5 SIVΔ*vpx* cases. One animal (case 4) that had 2 detectable SIV+ macrophages in LN out of 55 SIV+ cells also had the highest plasma viral load (**Table 3**) and two novel changes in Vif (L->P and A->T) (**Figure 2D** **)**. Of interest, is the observation that Ham-56+ myeloid cells with morphologic features of myeloid dendritic cells in the germinal centers were also negative for virus. Computer spectral imaging was used in order to discern spectrally distinct chromagens and to compare the frequencies of macrophage infection assessed by counting colabeled (yellow) cells on spectrally unmixed images (**Figure 6**).

**Table 3:** Phenotype of SIV+ cells in spleen, peripheral lymph node, and colon of SIVΔ*vpx*-infected monkeys by double-label *in situ* hybridization and immunohistochemistry.

Case #	Tissue	HAM56+/SIV+	CD3+/SIV+
1	LN	1/36	25/28
	SPL	0/16	19/23
	colon	0/27	ND
2	LN	1/49	51/53
	SPL	ND	ND
	colon	0/0	ND
3	LN	ND	ND
	SPL	0/4	2/2
	colon	0/0	ND
4	LN	2/55	47/56
	SPL	0/12	1/1
	colon	0/3	ND
5	LN	0/2	0/0
	SPL	0/5	7/7
	colon	ND	ND
	Total SPL/LN	4/179 (2.2%)	152/170 (89.4%)
	Total colon	0/30	ND
	TOTAL	4/209 (1.9%)	152/170 (89.4%)

We performed the same double labeling on colon sections from the 4 groups of animals (**Figure 5A**). There was a significant difference in the total and mean number of infected macrophages among the 4 groups (ANOVA p = 0.0023). The SIV239E (total 467/737, mean 66.7), SIV239noE (30/134, mean 9.8), and SIVΔ*nef*/SIVΔ3 groups (10/39, mean 3.3) had higher numbers of SIV+ macrophages in the colon compared to the SIVΔ*vpx* group, in which there were no SIV infected macrophages detected (all p<0.05) (**Figure 5C**). This translates into a much lower percentage of overall SIV+ cells that were macrophages in SIVΔ*vpx* rhesus (0%) compared to SIV239E (63.3%), SIV239noE (22.4%), and SIVΔ*nef*/SIVΔ3 groups (25.6%) (**Figure 5D**, **Table 4**). As observed in the lymphoid tissues of SPL/LN, the number of SIV+ macrophages in the colon from rhesus in the SIVΔ*nef*/SIVΔ3 group was significantly higher than in the SIVΔ*vpx* monkeys while there was no difference in the overall number of infected cells in these tissues between these 2 groups (**Figure 5** **B, C**).

**Table 4:** Summaryoflentiviral infection of macrophages in spleen, lymph node, anin macaques inoculated intravenously with SIVΔ*vpx* compared to SIVmac239 (with or without SIV encephalitis) and SIVΔ*nef* or Δ3 assessed in spleen, lymph node, and colon.

	Total # of SIV+ cells that were counted	Total # infected macrophages	Mean # SIV+ macrophages	% SIV+ cells that were macrophages
SPL and LN 239E	662	150	13.6	22.7%
SPL and LN 239noE	219	18	4.5	8.22%
SPL and LN Δ*vpx*	179	4	0.5*	2.2%*
SPL and LN Δ*nef* or Δ3	287	19	4.3	10.1%
Colon 239E	737	467	66.7	63.3%
Colon 239noE	134	30	9.8	22.4%
Colon Δ*vpx*	32	0	0.00025*	0%*
Colon Δ*nef* or Δ3	39	10	3.3	25.6%

*p<0.05 difference between SIVΔ*vpx* and both SIVmac239 and SIVΔ*nef*/Δ3cases.

The surprising scarcity of SIV-infected cells and complete absence of SIV+ macrophages (0/32 SIV+ cells) in the colon of the SIVΔ*vpx* monkeys highlights a potential role of Vpx and macrophage tropism *in vivo*, reflected in a difference in overall tissue tropism of SIVΔ*vpx* with an apparent reduction of virus trafficking to the colon. The one SIVΔ*vpx*-infected monkey (case 1) with more frequent SIV+ cells in the colon had severe bacterial enterocolitis, which likely contributed to enhanced immune activation and immune cell trafficking facilitating viral transport to the intestinal tract.

## Discussion

In this study, we have demonstrated that SIVΔ*vpx* is unable to efficiently infect tissue macrophages *in vivo*. Although SIVΔ*vpx*-infected animals had prolonged survival, they did eventually succumb to AIDS with CD4 decline despite the fact that there was little to no infection of macrophages [20]. *Vpx* enables infection of myeloid cells, including macrophage and dendritic cells, with HIV-2 and SIVmac viruses by inhibiting the recently identified macrophage restriction factor SAM domain HD domain-containing protein 1 (SAMHD1) [14]. Vpx loads the SAMHD1 protein onto the CRL4^DCAF1^ E3 ubiquitin ligase so that SAMHD1 undergoes proteaosome degradation allowing the virus to replicate [14]. As a consequence of the deletion of *vpx* in SIV, only 3 of the 5 SIVΔ*vpx*-infected macaques exhibited rare SIV+ macrophages in lymphoid tissues while the other 2 animals exhibited no macrophage infection. We also observed that SIVΔ*vpx*-infected animals had little to no virus in the colon despite the fact that there were target lymphocytes and macrophages present, and there was a complete absence of detectable SIV+ macrophages in the colon of any of the SIVΔ*vpx*-infected animals. In contrast, macrophage infection in cloned SIVmac239-infected rhesus occurred at significantly higher rates, especially in those cases that developed encephalitis. As an additional comparison group we analyzed tissues from rhesus infected with SIVΔ*nef* or SIVΔ3. Although tissue virus burden in SPL/LN and colon from the SIVΔ*nef* or SIVΔ3 animals were comparable to that of the SIVΔ*vpx*-infected animals, the number of SIV+ tissue macrophages and percentage of SIV+ cells that were macrophages were significantly lower in the SIVΔ*vpx* group. Nevertheless, all of the SIVΔ*vpx*-infected animals developed AIDS. It has long been postulated that HIV evolves broader cellular tropism from CD4+ T lymphocytes to macrophages and that this viral adaptation is necessary for progression to AIDS [31]. Our findings suggest that macrophage tropism is not necessary for development of immunosuppression and AIDS, but may be critical for dissemination of virus to the intestinal tract.

The profound and prolonged deficit in the ability of the *vpx*-deleted SIV to replicate in macrophages and dendritic cells in monkeys is consistent with the *in vitro* data on Vpx function but was not necessarily an obvious outcome. Wide ranges of culture conditions have been used for measuring the replication of SIV and HIV in macrophages *in vitro*. Both monocyte-derived macrophages obtained from peripheral blood and bronchoalveolar macrophages obtained by lung lavage have been used. Stimulating agents such as granulocyte-macrophage colony stimulating factor (GM-CSF) and macrophage colony stimulating factor (M-CSF) are used by some investigators but not others. These stimulants can have a dramatic effect on the ability of virus to replicate in macrophage cultures [32]–[34]. Dramatic differences in the ability of SIVmac239 and the SIVmac239-derived strain SIVmac316 to replicate in cultured macrophages are not necessarily reflected in their ability to replicate in monkeys experimentally infected with these strains [35]. In addition, SIV has a remarkable ability to evolve, to adapt, and to optimize its replication. Certainly this is demonstrated by the evolution of drug-resistant, neutralizing antibody-resistant, and CTL-resistant strains of SIV and HIV in an *in vivo* setting. Serra-Moreno, et al. have recently demonstrated that a *nef*-deleted strain of SIV adapted in monkeys to the loss of anti-tetherin activity by establishing such activity through mutation of a completely different region of the viral genome, the cytoplasmic domain of the gp41 transmembrane envelope glycoprotein [36]. The absence of any reasonable levels of SIVΔ*vpx* replication in macrophages in monkeys, even after two to three years of ongoing viral replication to provide an opportunity to adapt, even with the possibility that select pressure to restore this function may be limited, suggests that it is not easy for the virus to re-acquire the macrophage-specific *vpx* functions lost by its deletion.

Monkeys infected with SIVΔ*vpx* had lower viral loads and a slower progression to AIDS than what is typically observed with the parental SIVmac239, but nonetheless progressed to simian AIDS with low CD4 counts and AIDS-defining lesions. Two of the five SIVΔ*vpx*-infected monkeys that died with AIDS had no detectable infection of macrophages in spleen, lymph node or colon despite the presence of infected T cells in these animals at this terminal stage. Analysis of the other three monkeys revealed a total of four infected macrophages that were found. This dearth of infected macrophages contrasts markedly with all 11 control monkeys infected with parental SIVmac239. The frequency with which SIV encephalitis occurs with SIVmac239 in monkeys (about 30%) and the frequency of infected cells that are macrophages at a terminal stage appear to be similar to what is seen in HIV-1-infected people [23], [37], [38].

What might be the explanation for the few infected macrophages that were observed? As with any post-entry restriction factor, it is likely that there is a significant cell-to-cell variation in the amounts of SAMHD1 present in any individual macrophage or in different monocyte-macrophage subtypes. Some rare macrophages may end up with particularly low levels of SAMHD1 based on stage of differentiation, source, history, and activation state and these may be weakly susceptible to infection by *vpx*-deleted SIV. Alternatively, SAMHD1's ability to restrict infection may be down regulated in some cells. As with monkeys infected with *nef*-deleted SIV, in which another SIV gene (*env*) evolved the capacity for anti-tetherin activity [39], it is also possible that after many months *in vivo* the *vpx*-deleted SIV managed to evolve some anti-SAMHD1 activity in another SIV gene. In our limited sequence analysis there were no consistent or highly unusual, focused changes in the *vpr* or *tat* genes at the terminal stages in these animals to suggest what such change might be, although there were two novel changes in *vif*.

Findings in the colon are interesting and potentially informative. It is now clear that both SIV and HIV-1 cause extremely severe, sustained depletion of CD4+ CCR5+ T lymphocytes in gut-associated lymphoid tissue (GALT), including the colon. Severe depletion of this population of cells has been suggested to drive the evolution to CXCR4-using HIV-1 late in the course of disease [40]. It is also conceivable that this depletion of CD4 cells may drive selection of strains more competent for replication in macrophages. Breed et al (2013) recently reported that a virus with a 6 nucleotide deletion from the cytoplasmic tail of SIVmac239 Env (ΔGY) failed to result in depletion of CD4+CCR5+ cells in the gut lamina propria and also did not infect macrophages [41]. It has also been suggested that evolution of highly macrophage-competent strains of SIV appears late in the course of disease [24] and monkeys that survive the acute course of a severely CD4-depleting strain of SHIV exhibit almost exclusive infection of macrophages in lymphoid tissues many months later [42]. In the monkeys described here with SIV encephalitis, which had clearly evolved highly-macrophage-competent SIV, greater than 60% of the infected cells in the colon on average were macrophages. The numbers were lower in SIVmac239-infected monkeys that died without encephalitis, but they are still impressively high. What is remarkable is the near complete absence of infected macrophages in colon in the SIVΔ*vpx*-infected monkeys. Not only were macrophages not infected, but also T cells were rarely infected. In fact, there was a near complete absence of detectable virus in the colon of the SIVΔ*vpx*-infected monkeys. Perhaps infection of macrophages or dendritic cells is important for promulgating the infection of the small numbers of CD4+ CCR5+ lymphocytes remaining in the gut. This supports the recent *in vitro* findings that Vpx, by inducing SAMHD1 degradation, is important for HIV-1 transmission between T cells and dendritic cells, which is important for overall viral dissemination and persistence [17], [18].

In summary, we have shown that *vpx* is required for efficient myeloid cell infection in experimentally infected rhesus monkeys and that progression to AIDS and death can occur in the absence of Vpx and macrophage infection. This is interesting in light of the recent revelation that Vpx targets SAMHD1 for degradation [12]–[14], resulting in increased available dNTPs in macrophages and dendritic cells as substrates for lentiviral replication [8], [9]. Given this demonstrated role of Vpx in SIV-macrophage tropism, it is reasonable to think that a similar role is played by another viral protein in HIV-1, which does not encode Vpx. Surprisingly, HIV-1 Vpr, encoded by *vpr*, the paralog of *vpx*, does not inhibit or antagonize SAMHD1 while other primate Vpr proteins do [43]. From recent phylogenetic studies it is clear that the ability of primate lentiviruses to degrade SAMHD1 actually predated the origin of Vpx and was attributed to a neofunctionalization of Vpr [43]. Evidence of Vpr's ability to initiate degradation of SAMHD1 exists in several primate lentiviruses, but not in HIV-1 [43]. Since HIV-1 Vpr does not elicit degradation of human SAMHD1, there may be another viral protein or a different viral mechanism of HIV-1 that antagonizes SAMHD1 in order to enable macrophage and dendritic cell infection.

## Materials and Methods

### Ethics Statement

All animal studies were performed in accordance with federal laws and regulations, international accreditation standards, and institutional policies, including approval by the Institutional Animal Care and Use Committee of Harvard Medical School. All animals received two nutritional meals a day and fresh water ad libitum. All animals received required and approved environmental enrichment and were monitored daily for evidence of disease and changes in attitude, appetite, or behavior suggestive of illness. The enrichment program includes weekly rotation of enrichment devices including a wide variety of manipulanda, foraging feeders, sensory enrichment, grooming devices, destructible enrichment, and enrichment food items. Animals are socially housed except when this interferes with a study. Additional enrichment is targeted to animals that are assessed as having abnormal behavior or alopecia. Animals housed in the biocontainment facilities received a daily health check by both animal care technicians and veterinary professional staff. Appropriate clinical support was administered under the direction of the attending veterinarian and included analgesics, antibiotics, intravenous fluids, and other supportive care as needed. To ameliorate pain and suffering, all procedures were performed under ketamine, Telazol, or an inhalant anesthesia. Analgesics, such as buprenex, were used preemptively and following each potentially painful procedure. Animals were humanely euthanized when they presented with advanced stages of AIDS; criteria for euthanasia included 15% weight loss in two weeks, unresponsive opportunistic infection, persistent anorexia, intractable diarrhea, progressive neurologic signs, significant cardiac or pulmonary signs or other serious illness. The Harvard Medical School animal management program is accredited by the Association For Assessment and Accreditation of Laboratory Animal Care (AAALAC) and meets National Institutes of Health standards as set forth in “The Guide for the Care and Use of Laboratory Animals” (National Academy Press, 1996). The institution also accepts as mandatory the PHS “Policy on Humane care and use of Laboratory Animals by Awardee Institutions” and the NIH “Principles for the Utilization and Care of Vertebrate Animals used in Testing, Research and Training.”

### Animal cohorts, viral inocula, and clinical summary

The infectious pathogenic SIVmac239 clone [44], the *vpx*-deleted derivative of it called SIVΔ*vpx* [19], and the nef-deleted derivative called SIVΔ*nef* [45] have been described previously. SIVΔ3 is missing nef, vpr, and upstream sequences in U3 [22]. Five adult, Indian origin, rhesus macaques had been inoculated intravenously with a *vpx* deletion mutant virus based on parental cloned SIVmac239 (SIVΔ*vpx*) as previously described [19], [20]. Complete necropsies with tissue collection had been performed immediately following euthanasia of all monkeys. Formalin-fixed paraffin-embedded (FFPE) tissues were sectioned, stained with hematoxylin and eosin (H&E), and examined for the presence of macrophage-associated disease including multinucleated giant cell disease (GCD) and SIV encephalitis (SIVE). In addition, AIDS-associated lesions and diseases were evaluated and recorded for SIVΔ*vpx*-infected monkeys (**Table 2**). Archived tissues from adult, Indian-origin, rhesus macaques inoculated intravenously with cloned SIVmac239 with AIDS (n = 11), SIVΔ*nef* (n = 4), or SIVΔ3 (n = 2) were also evaluated for comparison (**Table 1**). The monkeys with AIDS were selected to represent a spectrum of disease induced by SIVmac239 and were included based on survival and progression to AIDS (including rapid and normal progressors), development of histologic features and opportunistic infections representative of SIVmac239-infected rhesus, and severity of encephalitis status, indicative of macrophage involvement. The diagnosis of SIV encephalitis was determined based on the presence of perivascular collections of SIV-infected macrophages and multinucleated giant cells in brain sections. Seven cases were diagnosed with SIV encephalitis (SIV239E, n = 7) while the remaining four monkeys lacked encephalitic changes (SIV239noE, n = 4).

All animal studies were performed in accordance with federal laws and regulations, international accreditation standards, and institutional policies, including approval by the Institutional Animal Care and Use Committee of Harvard Medical School. Animals were humanely euthanized when they presented with advanced stages of AIDS; criteria for euthanasia included 15% weight loss in two weeks, unresponsive opportunistic infection, persistent anorexia, intractable diarrhea, progressive neurologic signs, significant cardiac or pulmonary signs or other serious illness.

### Plasma viral RNA

Plasma viral RNA from SIVΔ*vpx*-infected rhesus macaques were tested by quantitative RT-PCR on stored plasma samples from chronic disease or near-terminal time points (range 329-1140 dpi, median 730 dpi) using techniques as previously described with some modifications [46]. Plasma samples harvested from whole blood collected in heparinized tubes required specialized techniques to purify RNA for real-time RT-PCR analysis. Briefly, viral RNA was purified using a BioSprint 96 One-For-All Vet Kit (QIAGEN, Switzerland, Germany, USA) following the manufacturer's recommendations for reagent volumes and elution conditions but processed manually using a Dynal MPC®-96S magnetic particle concentrator (LifeTechnologies, Grand Island, N.Y.). This kit purifies RNA by selective binding and release from silica-paramagnetic particles, eliminating heparin contamination that would inhibit RT-PCR. The yield recovery of RNA with this method is comparable to that from the standard method used in our laboratory [46] (Piatak, et al, unpublished).

### Cloning and sequencing of viral genes from SIVΔ*vpx*-infected rhesus plasma

Viral RNA was isolated from the plasma samples collected from the infected animal using High Pure Viral RNA Kit (Roche), reverse transcribed with SuperScript III One-Step RT-PCR System with Platinum Taq High Fidelity (Invitrogen). SIV sequences were amplified by polymerase chain reaction using Superscript III kit (Invitrogen) with S-VVout.s (5′-AAAAGGGTGGCTCAGTACTTATGCAGT-3′; positions 5829–5857 in SIVmac239 sequence) and S-VVout.as (5′-GATAAGCAGCTGATTCCCAAGACAT-3′; positions 6865–6889) for 40 cycles with primer annealing at 58oC for 30″ and primer extension at 68oC for 1′20″. PCR fragments were cloned directly into pCR2.1 vector using TOPO TA Cloning kit (Invitrogen) and inserts sequenced on both strands with S-VxVp.s (5′-ACTGCATAGCACTTATTTCCCTTGCT-3′; positions 5919–5944) and S-VxVp.as (5′-ATGCAGAAGATGTATTAGCCTTAGCCTTT-3′; positions 6818–6846) primers at CSHL shared DNA sequencing resource. The *vpx-vpr* region was amplified and sequenced successfully from plasma samples available from the SIVΔ*vpx* -infected rhesus macaques. Viral *vpx* gene sequences were analyzed and compared to parental viral clone SIVmac239. Predicted amino acid sequences for Vpx, Vpr, and overlapping Vif and Tat segments were constructed, aligned, and compared to parental viral clone SIVmac239. Alterations in amino acid sequences were searched against existing NCBI databases to determine novel mutations.

### *In situ* hybridization

The amount of SIV RNA in tissues was measured in spleen, peripheral lymph nodes, and colon (all when available) from each infected macaque in FFPE sections by *in situ* hybridization because of its high sensitivity [47]. Tissue sections were rehydrated in graded ethanol to 1X PBS made with diethyl pyrocarbonate (Sigma, St. Louis, MO) treated water. Endogenous alkaline phosphatase activity was blocked with levamisole (Sigma). Tissue sections were hydrolyzed in HCl (Sigma), digested with proteinase K (Roche Diagnostics, Indianapolis, IN), acetylated in acetic anhydride (Sigma), and hybridized overnight at 50°C with a digoxigenin-labeled antisense riboprobe which spans the entire genome of the molecular clone SIVmac239 (Lofstrand Labs, Gaithersburg, MD). Tissue sections were washed extensively and bound probe was detected using alkaline phosphatase-conjugated sheep anti-digoxigenin F(ab) fragments (Roche) and the chromogen nitroblue tetrazolium/5-bromo-4-chloro-3-indolyl-phosphate (NBT/BCIP, Roche). Sections were counterstained with Vector nuclear fast red (Vector Labs, Burlingame, CA). Sections of brain from a rhesus macaque with SIV encephalitis served as both positive control (when incubated with SIV antisense probe) and negative control (when reacted with SIV sense probe).

### Double-label *in situ* hybridization and immunohistochemistry of infected cells

Infected cells were characterized by double-label analysis *in situ* hybridization for SIVmac and immunohistochemistry with either anti-CD3 for T lymphocytes or anti-Ham56 for monocyte/macrophage lineage cells. *In situ* hybridization was performed as detailed above followed by routine immunoperoxidase staining on 5-µm formalin-fixed paraffin-embedded sections using standard avidin-biotin peroxidase complex techniques (Dako, Carpinteria, CA) as previously described [48], [49]. Briefly, sections were incubated with anti-human CD3 (rabbit polyclonal, Dako) or anti-human Ham56 antibody (Dako). Sections stained with anti-CD3 antibody were incubated with biotinylated goat anti-rabbit secondary antibody (Vector), and sections stained with anti-Ham56 antibody were incubated with biotinylated horse anti-mouse secondary antibody (Vector). Tissue sections were washed, developed with DAB chromogen (Dako), and counterstained with Vector nuclear fast red. Isotype-matched irrelevant controls were included for all runs.

### Quantification and Spectral Imaging

Sections were evaluated from each animal to derive a mean number of CD3+ lymphocytes and Ham56+ macrophages and SIV+ cells per 20× microscopic field as previously described [50], [51]. Briefly, positive cells were counted in 10 non-overlapping microscopic fields (a minimum of 100 cells or as many cells as possible). Due to the low number of SIV+ cells in SIVΔ*vpx*, SIVΔ*nef*, and SIVΔ3 cases, the entire slide was examined and all positive cells were counted for each case. All counting tasks were performed by two individuals blinded to the original of the slides. For double-label immunohistochemistry and *in situ* hybridization, sections were evaluated to assess percentage of infected cells that were macrophages per slide using a Nuance™ liquid crystal tunable filter based camera and multispectral imaging system (Caliper Life Sciences, Hopkinton, MA) interfaced with a Leica DMRE microscope. Images of tissue sections were captured without manipulation and analyzed using Nuance software. When there were fewer than 100 SIV+ cells the entire section was screened. Control slides of the following chromogens were used to make spectral libraries at each relevant magnification: diaminobenzidine (DAB) for immunohistochemistry, BCIP/NBT (*in situ*), and Vector Nuclear Fast Red (counterstain). The spectral absorbance of each chromogen was established from single stain controls and the contribution of each stain calculated from this reference to determine the relative contribution of the stain at each pixel. Images were automatically acquired from 420 nm to 720 nm at 20 nm steps and spectral components unmixed. Brightfield images were unmixed into a composite image and individual component images for each spectrum analyzed. With different chromogens unmixed, the numbers of SIV/Ham56 double-positive macrophages and SIV/CD3 double-positive T-lymphocytes were quantified in tissue sections and reported as percent of infected cells.

Generation of colocalization images was performed by overlaying masks of positive staining cells from the component images. A standardized threshold of minimum number of pixels determined each component mask. Simulated fluorescence images were converted from unmixed brightfield composite images by the Nuance™ software and then combined into a single image with colocalized signals represented in yellow.

### Statistical analysis

Statistical evaluation and generation of graphs of counted cells from immunohistochemistry and *in situ* hybridization assays was performed with the GraphPad Prism program (La Jolla, CA). Kruskal-Wallis nonparametric analysis of variance (ANOVA) followed by Dunn's multicomparison test were used to assess statistical differences between groups for each test with statistical differences assumed for probability values of p<0.05. For significant ANOVA, nonparametric Mann-Whitney *t* tests were performed for comparisons of mean number of infected cells and percent of SIV+ cells that are macrophages between groups with statistical differences assumed for p<0.05.

## Supporting Information

Figure S1 **Complete predicted amino acid sequences for** ***Vpx*** **(S1a) and** ***Vpr*** **(S1b) derived from viral RNA sequences from plasma.** Cullin 4 E3 binding region, required for counteraction of SAMHD1, is specified in sequence alignment of *Vpx*.
(DOCX)
