# Supplementary material for: SIV Vpx Is Essential for Macrophage Infection but Not for Development of AIDS
Source: PLoS One. 2014 Jan 21;9(1):e84463. doi: 10.1371/journal.pone.0084463 (PMC3897363; doi:10.1371/journal.pone.0084463)
Supplement: Figure S1 — Complete predicted amino acid sequences for Vpx (S1a) and Vpr (S1b) derived from viral RNA sequences from plasma. Cullin 4 E3 binding region, required for counteraction of SAMHD1, is specified in sequence alignment of Vpx. (DOCX) [file pone.0084463.s001.docx]

**Supplemental data**

**Fig. S1a**

***Vpx***

Required for binding

CRL4^DCAF1^ E3 Ub ligase

mac239 MSDPRERIPPGNSGEETIGEAFEWLNRTVEEINREAVNHLPRELIFQVWQRSWEYWHDEQGMSPSYVKYRYLCLIQKALFMHCKKGCRCLGEGHGAGGWRPGPPPPPPPGLA*

1-2 .......................*...................................*--------------------------------*...................*

1-3 .......................*................L...............QTSINGRKTSRK*

1-4 .......................*...................................*--------------------------------*...................*

1-5 .......................*.............S.................G...*--------------------------------*...................*

1-6 .....................L.*...................................*--------------------------------*...................*

1-7 .......................*........................*.G........*--------------------------------*...................*

1-8 .......................*................L...............QTSINGRKTSRK*

1-9 .......................*..............................*....*--------------------------------*...................*

1-10 .......................*...................................*--------------------------------*...................*

1-11 .......................*...................................*--------------------------------*...................*

1-12 .......................*........---------..................*--------------------------------*...................*

mac239 MSDPRERIPPGNSGEETIGEAFEWLNRTVEEINREAVNHLPRELIFQVWQRSWEYWHDEQGMSPSYVKYRYLCLIQKALFMHCKKGCRCLGEGHGAGGWRPGPPPPPPPGLA

2-3 .....V.................*................................QTSINGRKTSRK*

2-4 .......................*........-------------...*..........*--------------------------------*...................*

2-7 ................R......*...................................*--------------------------------*...................*

2-9 .......................*.............S.....................*--------------------------------*Y..................*

2-10 R......................*...................................*--------------------------------*...................*

2-14 ......-----............*........................*.G........*--------------------------------*...................*

2-15 ......-----............*...................................*--------------------------------*...................*

2-18 .......................*...................................*--------------------------------*...................*

2-20 I......................*...................................*--------------------------------*...................*

mac239 MSDPRERIPPGNSGEETIGEAFEWLNRTVEEINREAVNHLPRELIFQVWQRSWEYWHDEQGMSPSYVKYRYLCLIQKALFMHCKKGCRCLGEGHGAGGWRPGPPPPPPPGLA

3-1 ......K..............L.....................................*--------------------------------*...................*

3-2 ......K..............L.....................................*--------------------------------*...................*

3-3 ......K..............L....................................K*--------------------------------*...................*

3-4 ..G...K..............L.....................................*--------------------------------*...................*

3-7 ......K..............L.....................................*--------------------------------*...................*

3-9 ......K..............L*....................................*--------------------------------*...................*

3-10 ......K..............L.....I...............................*--------------------------------*...................*

3-11 ......K..............L.....................................*--------------------------------*...................*

3-18 ......K..............L.....................................*--------------------------------*...................*

3-19 ......K..............L.....................................*--------------------------------*...................*

3-20 ......K..............L.....................................*--------------------------------*...................*

3-24 ......KN.............L.....................................*--------------------------------*...................*

3-25 ......K..............L.............T.......................*--------------------------------*...................*

mac239 MSDPRERIPPGNSGEETIGEAFEWLNRTVEEINREAVNHLPRELIFQVWQRSWEYWHDEQGMSPSYVKYRYLCLIQKALFMHCKKGCRCLGEGHGAGGWRPGPPPPPPPGLA

4-21 I....................L.....I...........................*...*--------------------------------*...........S.......*

4-23 I....................L.*...............................*..K*--------------------------------*...........S.......*

4-24 I..L.................L.*.........................L.....*...*--------------------------------*...........S.......*

4-25 I....................L.*...I....................*..........*--------------------------------*...............T...*

4-26 .....................L.*.........................L.....*...*--------------------------------*...........S.......*

4-29 ............K.........*.........................*..........*--------------------------------*...........S.......*

4-30 I..L.................L.*...............................*...*--------------------------------*...........S.......*

4-31 ................I....L.*........................*..........*--------------------------------*...........S.......*

4-32 .....................L.*...A...............................*--------------------------------*...........S.......*

4-33 I....................L...........................L.....*...*--------------------------------*...........S.......*

4-34 I....................L.................................*...*--------------------------------*...........S...L...*

mac239 MSDPRERIPPGNSGEETIGEAFEWLNRTVEEINREAVNHLPRELIFQVWQRSWEYWHDEQGMSPSYVKYRYLCLIQKALFMHCKKGCRCLGEGHGAGGWRPGPPPPPPPGLA

5-1 ......................................................*....*--------------------------------*...................*

5-2 R..L.......................................................*--------------------------------*............S......*

5-3 ...........................................................*--------------------------------*.............S.....*

5-4 ..........................S............................*...*--------------------------------*...................*

5-5 ...........................................................*--------------------------------*.......L....S......*

5-7 .....................L.....................................*--------------------------------*...................*

5-8 .....................L.....................................*--------------------------------*...................*

5-9 .........................D.................................*--------------------------------*............S......*

5-10 R..........................................................*--------------------------------*........R..........*

5-11 R.......................................S..................*--------------------------------*...................*

5-12 ...........................................................*--------------------------------*........R..........*

mac239 MSDPRERIPPGNSGEETIGEAFEWLNRTVEEINREAVNHLPRELIFQVWQRSWEYWHDEQGMSPSYVKYRYLCLIQKALFMHCKKGCRCLGEGHGAGGWRPGPPPPPPPGLA

6-13 ................A.........K................................*--------------------------------*...................*

6-14 ............................I......V..................--------------------------------------*...................*

6-18 ................A..................V.......................*--------------------------------*.....*.............Y

6-19 ......................................................--------------------------------------*.....*.............Y

6-21 ..................................................G........*--------------------------------*Y....*...S.........*

6-22 ...........................................................*--------------------------------*.........S.........*

6-23 ...S.............T...............G......................*..*--------------------------------*.........S......E..*

6-24 ......................................................--------------------------------------*.....*.............Y

**Fig. S1b**

*Vpr*

mac239 MEERPPENEGPQREPWDEWVVEVLEELKEEALKHFDPRLLTALGNHIYNRHGDTLEGAGELIRILQRALFMHFRGGCIHSRIGQPGGGNPLSAIPPSRSML*

1-2 ....................A...................................................Y............................*

1-3 .......................................................................................A.............*

1-4 ..................................................................................................G..*

1-5 .....................................................................................................*

1-6 .....................................................................................R...............*

1-7 .....................................................................................................*

1-8 .....................................................................................................*

1-9 ...I.................................................................................................*

1-10 ......G...S..........................................................................................*

1-11 ......G..RS..........................................................................................*

1-12 .....................................................................................................*

mac239 MEERPPENEGPQREPWDEWVVEVLEELKEEALKHFDPRLLTALGNHIYNRHGDTLEGAGELIRILQRALFMHFRGGCIHSRIGQPGGGNPLSAIPPSRSML*

2-3 .........R........................................................................................G..*

2.4 .....................................................................................................*

2-7 ......G...S..........................................................................................*

2-9 ....................M................................................................................*

2-10 .....................................................................................................*

2-14 ..................................................................................................G..*

2-15 ................G....................................................................................*

2-18 ....................A...................................................Y............................*

2-20 ..................................................................................................G..*

mac239 MEERPPENEGPQREPWDEWVVEVLEELKEEALKHFDPRLLTALGNHIYNRHGDTLEGAGELIRILQRALFMHFRGGCIHSRIGQPGGGNPLSAIPPSRSML

3-1 ...................G...V..............W.I...............................Y............................*

3-2 ...................G...V..............W.I...............................Y.....................L...N..*

3-3 ...................G...V..............W.................................Y.....................L...N..*

3-4 ...................G...V..............W.................................Y..R..................L...N..*

3-7 ...................G...V..............W.................................Y.....................L...N..*

3-7 ...................G...V..............W.................................Y.....................L...N..*

3-10 ...................G...V..............W.................................Y.....................L...N..*

3-11 ...................G...V..............W.........S.......................Y.....................L...N..*

3-18 ...................G...V..............W.................................Y.....................L...N..*

3-19 ...................G...V..............W.................................Y.....................L...N..*

3-20 ...................G...V..............W.................................Y.....................L...N..*

3-24 ...................G...V..............W.I...........N...................Y.....................L...N..*

3-25 ...................G...V..............W.I...............................Y.....................L...N..*

mac239 MEERPPENEGPQREPWDEWVVEVLEELKEEALKHFDPRLLTALGNHIYNRHGDTLEGAGELIRILQRALFMHFRGGCIHSRIGQPGGGNPLSAIPPSRSML

4-21 .............................................Y............................S..................M....T..*

4-23 ...........L.................................Y................K...........S.......................N..*

4-24 ...........L...........M.....................Y............................S.................VT....N..*

4-25 ...........L.................................Y............................S.................VT....N..*

4-26 ..........................................................................S..................T....N..*

4-29 .....................K.................................................................R..........T..*

4-30 .............................................Y............................S.......................T..*

4-31 ...........L............K....................Y................K...........S..................T....N..*

4-32 .............................................Y............................S..................T....N..*

4-33 .............................................Y..............I.............S..................TQ...N..*

4-34 .............................................Y............................S.................VT....N..*

mac239 MEERPPENEGPQREPWDEWVVEVLEELKEEALKHFDPRLLTALGNHIYNRHGDTLEGAGELIRILQRALFMHFRGGCIHSRIGQPGGGNPLSAIPPSRSML*

5-1 ....................................................................................A................*

5-2 ....................................................................................A................*

5-3 ....................................................................................T................*

5-4 ....................................................................................A................*

5-5 ....................................................................................T................*

5-7 ....................................................................................T................*

5-8 ....................................................................................T................*

5-9 ....................................................................................A................*

5-10 ..........S.........................................................................A................*

5-11 ......................I.............................................................A................*

5-12 ..........S.........................................................................A................*

mac239 MEERPPENEGPQREPWDEWVVEVLEELKEEALKHFDPRLLTALGNHIYNRHGDTLEGAGELIRILQRALFMHFRGGCIHSRIGQPGGGNPLSAIPPSRSML*

6-13 .....................................................................................W...............*

6-14 .............................................Y.......................................................*

6-17 ............................K.........W..V...Y.......................................................*

6-18 .............................................Y............E..........................................*

6-19 .............................................Y.......................................................*

6-20 ......................................W..V...Y.......................................................*

6-21 ...................................H.SW..V...........................................................*

6-22 ..G..........................................Y.......................................................*

6-23 ......................................W..V...........................................................*

6-24 .............................................Y.......................................................*
